# Effect of end expiration breath hold on target volumes and organ at risk doses for oesophageal cancer radiotherapy

**DOI:** 10.1016/j.phro.2025.100726

**Published:** 2025-02-07

**Authors:** Christopher Mayhew, Jeyaanth Venkatasai, Marina Khan, Victoria Butterworth, Kasia Owczarczyk, Georgios Ntentas

**Affiliations:** a Department of Medical Physics and Clinical Engineering Guy’s and St Thomas’ NHS Foundation Trust London UK; b Department of Oncology Guy’s Cancer Centre Guy’s and St Thomas’ NHS Foundation Trust London UK; c Department of Radiotherapy Guy’s Cancer Centre Guy’s and St Thomas’ NHS Foundation Trust London UK; d Nuffield Department of Population Health University of Oxford Oxford UK; e School of Biomedical Engineering and Imaging Sciences King’s College London London UK

**Keywords:** Oesophageal cancer, End expiration breath hold, EEBH

## Abstract

**Background and Purpose:**

The end expiration breath hold (EEBH) technique has the potential to reduce tumour motion during radiotherapy treatment of lower oesophageal cancer, and therefore, motion artefacts, target volumes and dose to surrounding organs at risk (OAR). EEBH is an emerging technique and clinical data on its use in oesophageal cancer is scarce.

**Methods and Materials:**

A comparison of 20 lower oesophageal cancer patients was performed for radiotherapy treatment plans in both EEBH and free breathing (FB). EEBH and FB plans were evaluated and compared in terms of motion artefacts, target volumes and dose-volume metrics.

**Results:**

EEBH was effective in reducing the observed motion artefacts seen in planning CTs compared to FB. EEBH also significantly reduced the average PTV size between EEBH and FB (ΔV = -48 ± 55 cm^3^; p < 0.001). OAR-PTV overlap volumes were also effectively reduced in EEBH compared to FB, including for lung-PTV overlaps (ΔV = -13 ± 13 cm^3^; p < 0.001) and for heart-PTV overlaps (ΔV = -8 ± 14 cm^3^; p = 0.02). Mean heart doses were lower on average by −1.2 ± 2.0 Gy with EEBH (p = 0.02), and mean lung doses by −1.0 ± 1.0 Gy (p < 0.001). Mean liver doses were on average reduced with EEBH by −0.6 ± 1.5 Gy, whereas spinal D_2cm_3 increased in EEBH compared to FB by 1.8 ± 6.3 Gy, but neither were statistically significant.

**Conclusion:**

Use of EEBH for oesophageal cancer radiotherapy reduced motion artefacts and increased confidence in contouring volumes. Additionally, planning target volumes and doses to key OARs were reduced with EEBH compared to FB plans.

## Highlights

• End expiration breath hold is well tolerated by oesophageal cancer patients.
• End expiration breath hold reduced CT motion artefacts compared to free breathing.
• End expiration breath hold reduced target volumes and doses to organs at risk.

## 1 Introduction

Lower oesophageal cancer rates have been increasing in the United States and Europe over recent decades [1]. In addition, survival rates remain low due to the location of the disease with its proximity to other organs, and its likeliness to metastasise to other areas [2]. However, in the last 40 years, there has been a three-fold increase in the likelihood of ten-year survival from 4 % to 12 % due to advances in both diagnosis and treatment of the disease [3]. Radiotherapy plays a crucial role in the curative treatment of oesophageal cancer and is often delivered either as neoadjuvant chemoradiotherapy (prior to surgery) or as definitive chemoradiotherapy [4]. In patients unfit for chemotherapy, radiotherapy is used as a sole modality for local control of disease [5].

Advanced radiotherapy techniques, such as intensity modulated radiotherapy, volumetric modulated arc therapy and proton beam therapy, have helped further improve dose distributions by minimising high dose to the surrounding organs while delivering adequate dose to the target [6], [7], [8]. In oesophageal cancer radiotherapy, the degree to which breathing can affect the motion of the target volume and surrounding anatomy varies depending on the location of the tumour, the individual’s internal anatomy and breathing pattern [9]. Often, tumours in the lower oesophagus extending into the gastroesophageal junction (GOJ) suffer from this motion to a higher degree due to the proximity to the diaphragm [10]. This motion was quantified in a study by Doi et al. [11] which showed that superior-inferior motion was the most significant in the lower oesophagus. Due to the influence of breathing motion, the potential for breath holds to aid in reducing motion during treatment [9], and hence increase the accuracy of radiotherapy, is an area of great importance in lower oesophageal cancers.

The end expiration breath hold (EEBH) is characterised by the patient’s expulsion of air to their natural greatest extent, and it has the advantage of being more reproducible and stable than other types of breath holds such as deep inspiration breath hold (DIBH) [12], [13], [14]. It has demonstrated reductions in motion and target volume sizes, and therefore, potential for improving dose sparing of organs at risk (OAR) in the oesophageal region [15]. Furthermore, it has been found that tumour motion in the lower oesophagus is more significant than for mid and upper oesophageal cancers [16], [17]. This is especially prevalent in the anteroposterior (AP) and craniocaudal (CC) directions and emphasises the potential benefits that could be seen from EEBH in reducing internal motion. These findings agree with The Royal College of Radiologists’ 2021 report on updated guidance for image guided radiotherapy [18], which states that the lower oesophagus is the most mobile section of the oesophagus. However, EEBH presents limitations, such as potential reductions in the PTV-to-OAR distances as well as patients’ frequent difficulties in holding in EEBH compared to other breath holds, such as DIBH, which highlights the importance of evaluating its potential volumetric and dose-volume benefits [19]. Longer treatment times have also been reported for EEBH compared to FB, and as such EEBH must demonstrate a clinical benefit to justify its use [20].

In this study we compared motion artefacts between EEBH and FB scans for oesophageal cancer radiotherapy and the effects of EEBH in target volumes and dose-volume metrics for the main OARs compared to FB.

## 2 Materials and methods

### 2.1 Patient data and imaging protocols

In this study, we enrolled patients with existing clinical treatment plans who had received radiotherapy at our centre between June 2021 and February 2023. Data collection was performed retrospectively between June 2022 to December 2023. The inclusion criteria for this study were patients with lower oesophageal and GOJ tumours who had received radical (chemo)radiotherapy. These were identified from departmental radiotherapy records. For this study, only patients with both EEBH as well as 4DCT planning scans were included. Patients with both squamous cell carcinoma as well as adenocarcinoma and any TNM stage and tumour length were included. Exclusion criteria included excessive motion artefacts in patients’ average 4DCTs which resulted in the inability to accurately delineate the target volumes and OAR.

Patients had both an EEBH CT scan (for an EEBH treatment) and a 4DCT scan (for a FB treatment) on a Discovery CT590 RT GE CT Scanner (GE Healthcare, Chicago, IL). Scans were performed with a tube voltage of 120 kV and a slice thickness of 2.5 mm. Our clinical protocol required that for patients suitable for EEBH, an additional 4DCT was acquired in case the patient demonstrated an inability to hold in EEBH adequately and reproducibly within pre-set thresholds during their course of treatment using EEBH.

Motion artefacts were also assessed for both EEBH CTs and 4DCTs due to their impact on accurate structure delineation. Major motion artefacts were classed as causing uncertainty in delineation of OARs and target volumes. Minor artefacts were classed as being observable but not causing uncertainty in delineations.

Initially, 22 patients were identified for this study, with two eventually being excluded due to major motion artefacts in their average 4DCT. The excluded patients had been treated entirely in EEBH and as such, their 4DCT had not been used for planning. Nineteen of the remaining 20 patients had an existing clinical EEBH plan with which they began treatment. One patient started treatment in FB despite having an EEBH CT due to difficulties managing the breath hold. Two EEBH patients had re-plans during treatment using their 4DCT due to difficulties holding in EEBH as treatment progressed.

Patient characteristics are summarised in Table 1. Whilst most patients were diagnosed with adenocarcinoma (ADC), three patients had squamous cell carcinoma (SCC), while one had an undetermined type. The median age was 73 years, sixteen of the 20 patients were male. A range of radiotherapy prescriptions were used, ranging from 41.4 Gy in 23 fractions to 55 Gy in 20 fractions.

**Table 1:** Key patient characteristics of the study cohort.

**Age***(years)*	Median	73
	Minimum	54
	Maximum	87
**Sex***(freq.)*	Male	16 (80 %)
	Female	4 (20 %)
**Histology***(freq.)*	SCC	3 (15 %)
	ADC	16 (80 %)
	Other	1 (5 %)
**Treatment Intent***(freq.)*	Radical	15 (75 %)
	Preoperative	2 (10 %)
	Postoperative	3 (15 %)
**T Stage***(freq.)*	T1	0 (0 %)
	T2	2 (10 %)
	T3	16 (80 %)
	T4	2 (10 %)
**N Stage***(freq.)*	N0	2 (10 %)
	N1	6 (30 %)
	N2	11 (55 %)
	N3	1 (5 %)
**Radiation Prescription***(freq.)*	50 Gy/25#	12 (60 %)
	41.4 Gy/23#	2 (10 %)
	55 Gy/20#	1 (5 %)
	50.4 Gy/28#	5 (25 %)
**Concurrent Chemotherapy***(freq.)*	Yes	19 (95 %)
	No	1 (5 %)

SCC: squamous cell carcinoma, ADC: adenocarcinoma, T: tumour, N: Nodal, M: metastases.

#: fraction, Gy: Gray, freq: frequency.

A clinical audit request to investigate the use of EEBH in oesophageal patients retrospectively was submitted to the Guy’s and St Thomas’ Radiotherapy Operational Group and was approved.

### 2.2 End expiration breath hold Workflow

In the EEBH technique, patients were set up according to our centre’s local thoracic protocol. The Real-time Position Management (RPM) (Varian Medical Systems, Palo Alto, CA) was used to monitor the breathing cycle and ensure reproducibility of the breath hold during treatment. The RPM continuously tracked external motion, with a beam hold feature to enhance safety by interrupting the beam if the patient deviated from the breath hold within predefined thresholds established during the patient’s planning CT. During respiratory monitoring, the RPM system generated a graphical display of the patients’ breathing phases, allowing therapeutic radiographers to visualise respiratory patterns. Fiducial markers were not utilised.

For the EEBH, patients were instructed to breathe normally and then ‘stop’ in the exhalation phase. Local experience found this simple instruction facilitated more consistent, prolonged breath hold within the required thresholds than prior coaching. Breath hold coaching was provided for patients who required further guidance. The mode average of EEBHs required per fraction was eight (four per arc) with individual assessment for each patient.

A 3D cone beam CT (CBCT) was acquired following RPM confirmation that the patient was in EEBH within the required thresholds. The CBCT was used to assess patient positioning and the stability of the breath hold was determined through assessment of motion artefacts of the acquired image.

### 2.3 Target delineation and treatment planning

In this study, a retrospective treatment plan was created on the non-treated CT scan. Treatment planning was performed on the EEBH CT and the average 4DCT for the EEBH and FB plans respectively. EEBH PTVs were created by expanding the Clinical Target Volume (CTV) 10 mm superiorly & inferiorly and 5 mm circumferentially. FB PTVs were created by expanding the Internal Target Volume (ITV) 5 mm isotropically as per SCOPE 2 4D protocol [21]. The ITV was generated using 4DCT datasets generated from the corresponding phases of respiration.

Treatment planning methods followed the guidelines from the Study of Chemoradiotherapy in Oesophageal Cancer including PET Response and Dose Escalation (SCOPE2) trial, including target delineation and dose-volume constraints [21]. All treatment plans were planned in the Eclipse treatment planning system (TPS) with dose calculated with the AcurosXB v13.6 dose calculation algorithm. Treatments were delivered using TrueBeam Varian LINACs (Varian Medical Systems, Palo Alto, CA). Planning techniques were guided by the ESTRO RATING system for planning comparison studies to ensure the fair comparison between EEBH and FB plans for each patient [22]. This included the number of arcs, planning structure creation, comparable optimiser settings, and treatment planning algorithm. The primary points to ensure a comparable plan were 1) a PTV dose to > 95 % of the target volume (D_95%_) within ± 0.2 Gy from the clinical plan 2) a slice-by-slice assessment of the dose distribution between the FB and EEBH plans visually comparing the PTV and OARs dose coverage and magnitude of hot and cold spots 3) dose-volume histograms (DVHs) were compared and 4) both EEBH and FB plans were checked by a senior physicist and approved by a clinical radiation oncologist to ensure clinical acceptability.

Paired t-tests tests were used to compare volumes, mean organ doses and D_2cm3_ (for spinal cord) between EEBH and FB plans (Table 2, Table 3).

**Table 2:** Comparison of average target and OAR volumes for oesophageal cancer patients between EEBH and FB.

**Volume**	**Observations**	**EEBH (cm^3^)** **Average (Range)**	**FB (cm^3^)** **Average (Range)**	Difference* (cm^3^) **Average (Range)**	**P-Value**
PTV	20	436 (133–1246)	485(154–1219)	−48(−136 to 46)	<0.001
Heart	20	782 (455–1123)	771 (402–1061)	11 (−96 to 147)	0.39
Bowel	6	407 (108–946)	444 (158–739)	−37 (−122 to 241)	0.60
Liver	20	1524 (933–2639)	1315 (635–2026)	208 (0 to 613)	<0.001
Lung	20	3242 (2224–4520)	3829 (2591–5531)	−587 (−1803 to 390)	<0.001
Spleen	14	244 (80–506)	189 (24–447)	55 (−12 to 164)	0.003
Stomach	12	227 (84–547)	208 (78–606)	19 (−59 to 176)	0.36
Spinal	20	26 (13–45)	25 (13–52)	1 (−14 to 13)	0.62
Heart-PTV Overlap	20	31 (1–64)	40 (8–61)	−8 (–32 to 19)	0.02
Lung-PTV Overlap	20	16 (0–84)	29 (3–85)	−13 (−47 to 10)	<0.001

OAR: organ at risk, EEBH: end expiration breath hold, FB: free-breathing, PTV: planning target volume.

* EEBH – FB volumes.

**Table 3:** Comparison of average mean OAR doses for oesophageal cancer patients between EEBH and FB.

**Organ at risk;** (notation for dose)	**Observations**	**EEBH (Gy)** **Average (Range)**	**FB (Gy)** **Average (Range)**	Difference* (Gy) **Average (Range)**	**P-Value**
Heart (mean)	20	17.7 (5.5–26.3)	18.9 (8.8–26.2)	−1.2 (−4.0 to 2.6)	0.02
Bowel (mean)	6	11.6 (1.6–18.2)	11.4 (1.4–17.9)	0.1 (−1.5 to 2.0)	0.45
Liver (mean)	20	11.9 (1.2–17.8)	12.5 (2.4–20.1)	−0.6 (−3.2 to 2.6)	0.10
Lung (mean)	20	7.4 (2.0–14.0)	8.3 (3.2–15.1)	−1.0 (−2.4 to 1.0)	<0.001
Spleen (mean)	14	15.4 (8.8–27.3)	13.9 (0.8–26.5)	0.3 (−5.4 to 4.9)	1.00
Stomach (mean)	12	29.9 (10.3–39.0)	32.5 (2.1–41.3)	−2.7 (−15.3 to 4.4)	0.10
Spinal (D_2cm_^3^)	20	21.3 (9.0–34.1)	19.6 (11.3–34.6)	1.8 (−5.6 to 13.4)	0.11

OAR: organ at risk, EEBH: end expiration breath hold, FB: free-breathing, D_2cm_^3^: maximum dose to 2.0 cm^3^ of the structure, Gy: Gray.

*EEBH – FB doses.

## 3 Results

**Fig. 1:**
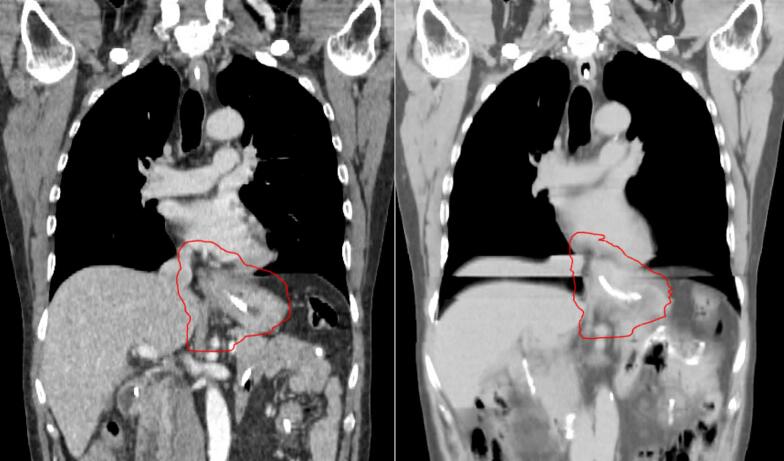
A) the end expiration breath hold ct and b) motion artefacts seen in the same coronal view of the 4dct for the same patient. planning target volume outlined in red. (For interpretation of the references to colour in this figure legend, the reader is referred to the web version of this article.)

Motion artefacts were observed in all 20 4DCTs. Eight 4DCTs contained major motion artefacts, and 12 contained minor motion artefacts (Fig. 1). The majority of these were observed in the region of the diaphragm and were especially prevalent for liver, lung, and bowel structures. This increased the difficulty in the delineation of these organs. Frequently, these motion artefacts were in the region of/or overlapping with the target volume’s inferior extent. No observable motion artefacts were seen in any EEBH CTs.

We found a significant reduction in PTV volumes with EEBH as compared with FB treatment with average volumes of 436 cm^3^ and 485 cm^3^, respectively, and an average difference of −48 ± 55 cm^3^ (p < 0.001). As expected, there was a significant reduction with EEBH in lung volumes with an average reduction of −587 ± 503 cm^3^ (Range: 1803 cm^3^ to 390 cm^3^; p < 0.001) and a significant increase in liver volumes with an average increase of 208 ± 139 cm^3^ with EEBH (Range: 0 cm^3^ to 613 cm^3^; p < 0.001). We further observed a significant reduction in lung and heart-PTV overlap volumes with EEBH (Table 2). Mean heart doses were reduced with EEBH on average by −1.2 ± 2.0 Gy compared to FB (Range: 4.0 Gy to 2.6 Gy; p = 0.02). Mean lung doses were also reduced with EEBH on average by −1.0 ± 1.0 Gy (Range: 2.4 Gy to 1.0 Gy; p < 0.001). There was no significant change in mean organ doses for liver, stomach, bowel, or spleen. Spinal D_2cm3_ was increased in EEBH compared to FB but not significantly (Table 3).

## 4 Discussion

This study covered a range of lower oesophageal tumours using clinical EEBH or FB treatment plans. We found that EEBH eliminated major motion artefacts compared to FB. Significant reductions in target and OAR volumes were found in EEBH compared to in FB. This resulted in significant reductions to OAR doses, including to heart and lungs. To our knowledge, this is the first study to compare EEBH to FB treatment plans for oesophageal radiotherapy in terms of motion artefacts as well as dose-volume metrics and volumetric changes.

The 4DCT motion artefacts increased difficulty in organ and target delineation, however, the uncertainties in the target delineation were accounted for in part by the ITV which accounts for associated errors due to motion. This was a clear advantage of EEBH and has been previously reported in Ehrbar et al. [23], which reported the negation of the ITV in EEBH and the increased ease of target delineation due to motion artefact reduction. A further potential benefit could be observed if consideration is given for reducing the PTV expansion for the EEBH PTV due to the motion reduction EEBH provides, for example, reducing the 10 mm margin superiorly & inferiorly to 5 mm.

Moreover, we observed that the image quality of the CBCT taken in EEBH during treatment was superior to that in FB, particularly for abdominal regions such as lower oesophagus, where motion can severely affect the quality of the verification images. Ehrbar et al. [24] reported not only the benefits to radiotherapy delivery with reduced motion artefacts but also potential improvements to image fusions and CBCT image quality. However, EEBH can cause longer treatment times due to the difficulties some patients faced to reproduce EEBH consistently. For that reason, the treatment appointments were longer than conventional FB appointments, where oesophageal EEBH and FB appointments were 30 min versus 15 min per patient respectively, which adds pressures and costs to an already busy radiotherapy department. In the last couple of decades, there has been a rise in the adoption of breath-hold techniques to address the challenges posed by organ motion during radiotherapy. Although the deep inspiratory technique is most commonly used due to its ease and motion reduction, expiratory breath hold was shown in our study and in a study on pancreatic cancer to be advantageous in upper abdominal tumours with increased motion reduction [12].

Volumetrically, EEBH showed promise in reducing the absolute volumes of some key OARs, PTVs, and overlapping volumes. This was in keeping with a prior study evaluating EEBH in stereotactic ablative radiotherapy in upper abdominal tumours (liver and pancreas) which showed a 31 % average reduction in target volume with EEBH [15]. The PTV volume reduction in our study was not unexpected, as due to the motion reduction, the creation of an ITV was not performed for EEBH. The PTV volume for the FB plans was, therefore, dependent on the extent of the movement in free breathing. Meanwhile, the EEBH PTV was created directly from the CTV. As such, it is likely that dose-volume metric differences seen between FB and EEBH will be due to the margin expansions. However, it is the magnitude of the stability of this breath hold in this region that justifies these expansions. Tahir et al. [25] also reported PTV volume reductions in EEBH, although compared to DIBH treatments for non-small cell lung cancer. Likewise, Biancia et al. [26] compared end inspiration to end expiration breath holds for lung cancer, finding reductions in both lung and PTV volumes.

Heart, bowel, stomach, and spinal volumes showed no statistically significant difference between EEBH and FB as expected which demonstrates consistent OAR delineations between scans. Lung volumes were significantly reduced with EEBH compared to FB, as expected, and the lung-PTV overlap volumes were also significantly reduced. On the other hand, liver volumes increased compared to FB, where the diaphragm pushes upon the liver. Liver-PTV overlap volumes were not always available as most PTVs in both EEBH and FB did not overlap, and as such, are left out of our analysis. Heart-PTV overlap volumes were also reduced in EEBH.

EEBH demonstrated an OAR dose advantage compared to FB treatments as it helped reduce mean doses to the heart and lungs significantly. This was more likely due to the PTV volume reduction due to the negation of the ITV and the resultant decrease of the total irradiated volume, and thus, heart and lung overlap volumes with the PTV. In general, radiotherapy to the oesophagus is associated with a substantial dose to the heart and lungs, and patients with oesophageal cancer typically present less favourable cardiovascular and pulmonary risk profiles [27], [28]. Therefore, any efforts to mitigate these cardiac risks and improve survival rates by reducing mean heart and lung doses should be considered. Additionally, recent retrospective work in oesophageal cancer patients reported that dose to the coronary artery was a better predictor of acute coronary syndrome than mean heart dose [29], therefore, contouring cardiac substructures and assessing EEBH’s dose benefits in those could provide a better insight of the potential benefits of this technique in cardiac risk sparring. Further studies performed prospectively with more patients and correlation with toxicity outcomes would clarify any relationship between EEBH and reductions of these organ doses and associated risks.

In terms of patient experience, we performed a local audit between 2018–2021 for patients deemed suitable for EEBH and found that 12 out of 51 patients (23.5 %) could not hold EEBH to the necessary criteria upon acquisition of the EEBH CT [30]. A further 3 (5.9 %) of these patients failed to hold in EEBH for the whole of their treatment. It is worth noting, however, that patients in the audit included a variety of treatment sites; 38 liver, 5 lower oesophagus, 2 pancreas and 5 abdominal node patients.

EEBH plans have demonstrated their effectiveness in reducing motion artefacts compared to FB plans. These motion reductions, as well as the change in the internal anatomy, reduces key OAR volumes and overlap volumes compared to the FB plan. In many cases, these reductions do translate into OAR mean dose reductions in EEBH. Our study demonstrated volumetric and OAR dose advantages of EEBH over FB treatments in lower oesophageal cancer radiotherapy. Whether this, in turn, will translate into a clinical benefit in terms of toxicity reduction and improved local control to justify the additional time and cost will need to be assessed in a larger prospective clinical series.

## Funding

Dr Ntentas was supported by a National Institute of Health Research Clinical Lectureship (NIHR301261) funded by Health Education England (HEE) and by 10.13039/501100000289Cancer Research UK (C8225/A21133, PRCRPG-Nov21\100001) and the University of Oxford. The views expressed are those of the authors and not necessarily those of the NIHR or the Department of Health and Social Care or Cancer Research UK.

## CRediT authorship contribution statement

**Christopher Mayhew:** Conceptualization, Methodology, Formal analysis, Investigation, Writing – original draft, Writing – review & editing. **Jeyaanth Venkatasai:** Resources, Writing – original draft, Writing – review & editing. **Marina Khan:** Resources, Writing – original draft, Writing – review & editing. **Victoria Butterworth:** Writing – review & editing, Visualization. **Kasia Owczarczyk:** Resources, Writing – original draft, Writing – review & editing. **Georgios Ntentas:** Conceptualization, Methodology, Formal analysis, Writing – original draft, Writing – review & editing, Funding acquisition.

## Declaration of competing interest

The authors declare that they have no known competing financial interests or personal relationships that could have appeared to influence the work reported in this paper.
